# Draft Genome Sequence of Mycolicibacterium sp. Strain CH28, a Potential Degrader of Diisopropyl Ether, Isolated from Pharmaceutical Wastewater

**DOI:** 10.1128/MRA.00682-19

**Published:** 2019-09-12

**Authors:** Ingrid Zsilinszky, Péter Gyula, Zoltán Bihari, Balázs Fehér, Zsolt Szabó

**Affiliations:** a Department of Applied Microbiology, Division for Biotechnology, Bay Zoltán Nonprofit Ltd. for Applied Research, Szeged, Hungary; b Department for Plant Biotechnology, National Agricultural Research and Innovation Centre, Gödöllő, Hungary; c Xenovea Ltd., Szeged, Hungary; University of Southern California

## Abstract

Mycolicibacterium sp. strain CH28 is a novel bacterial isolate belonging to a group of rapidly growing mycobacteria. Here, we report the draft genome sequence of strain CH28 and provide insights into the genetic background of its potential diisopropyl ether-degrading capability.

## ANNOUNCEMENT

Rapidly growing mycobacteria are free-living saprophytes that are widely distributed in various natural habitats (1). Some of these species exhibit a high capacity for the biodegradation of a broad range of environmental pollutants, including several dialkyl ethers (1–3).

Diisopropyl ether (DIPE) is an extensively used industrial solvent and fuel additive; thus, it can be a major pollutant in aquatic environments. To date, only three DIPE-degrading bacterial isolates have been described, Rhodococcus ruber IFP 2001 (4), Pseudonocardia sp. strain ENV478 (5), and Aquincola tertiaricarbonis L108 (6). Hence, the genes and enzymes involved in DIPE biodegradation are only partially known (7).

Mycolicibacterium sp. strain CH28 was isolated from a wastewater sample collected at a pharmaceutical production facility in Budapest, Hungary. Approximately 10 ml of the sample was inoculated into 90 ml of mineral salts medium (MSM) (8), and 2 mM DIPE was added as the enrichment substrate. After 10 days of incubation at 25°C with shaking at 150 rpm, 1 ml culture was transferred into 100 ml fresh MSM and incubated under the same conditions with 2 mM DIPE. After the third consecutive transfer, the enrichment culture was serially diluted and plated onto MSM agar plates containing 2 mM DIPE. One pure strain, designated CH28, was isolated and chosen for further study.

To investigate the genetic background of the potential DIPE-degrading ability in strain CH28, we sequenced its genome. Genomic DNA was extracted from a single colony grown on MSM agar with 2 mM DIPE at 25°C using an UltraClean microbial DNA isolation kit (MO BIO Laboratories, Inc.) according to the manufacturer’s instructions. The genomic library was prepared with a SureSelect^QXT^ reagent kit (Agilent) and sequenced on an Illumina MiSeq platform with 250-bp paired-end chemistry. The raw sequences were corrected with Lighter (v1.1.1) (9), merged with FLASH (v1.2.11) (10), and decontaminated with DeconSeq (v0.4.3) (11), by using the UniVec database (v10.0) as a reference. The unpaired reads were resynchronized with fastq-pair (v0.1) (12). The clean reads were assembled with MIRA (v5rc2) (13). The complete workflow can be found at https://github.com/gyulap/CH28_genome_assembly.

A total of 1,214,702 reads were assembled, resulting in 43 contigs (longer than 500 bp) with a total length of 6,046,830 bp (48× coverage). The *N*_50_ value was 426,226 bp. The genome has an average G+C content of 66.7%. The sequences were annotated using the NCBI Prokaryotic Genome Annotation Pipeline (14).

Genome sequence data revealed the presence of EthB (15), showing 99% similarity to the corresponding proteins of *R. ruber* IFP 2001 and *A. tertiaricarbonis* L108. This cytochrome P450 monooxygenase was experimentally proved to be responsible for the degradation of DIPE, along with other dialkyl ethers (4, 6, 15). Its regulator, EthR, was also detected, suggesting that the *eth* operon is under specific regulation in *Mycolicibacterium* sp. strain CH28.

### Data availability.

This whole-genome shotgun project has been deposited in DDBJ/ENA/GenBank under accession no. SRLQ00000000 (BioProject accession no. PRJNA520790; BioSample accession no. SAMN10867835). The version described in this paper is the first version, SRLQ01000000. The raw sequences have been deposited in the NCBI SRA database under accession no. SRR8797416.

## References

[1] Hartmans S, de Bont JAM, Stackebrandt E 2006 The genus *Mycobacterium*—nonmedical, p 889–918. *In* Dworkin M, Falkow S, Rosenberg E, Schleifer K-H, Stackebrandt E (ed), The prokaryotes, 3rd ed, vol 3 Springer, New York, NY.

[2] François A, Mathis H, Godefroy D, Piveteau P, Fayolle F, Monot F 2002 Biodegradation of methyl *tert*-butyl ether and other fuel oxygenates by a new strain, *Mycobacterium austroafricanum* IFP 2012. Appl Environ Microbiol 68:2754–2762. doi:10.1128/aem.68.6.2754-2762.2002. 12039730 PMC123982

[3] Lopes Ferreira N, Maciel H, Mathis H, Monot F, Fayolle-Guichard F, Greer CW 2006 Isolation and characterization of a new *Mycobacterium austroafricanum* strain, IFP 2015, growing on MTBE. Appl Microbiol Biotechnol 70:358–365. doi:10.1007/s00253-005-0074-y. 16028043

[4] Hernandez-Perez G, Fayolle F, Vandecasteele J-P 2001 Biodegradation of ethyl *t*-butyl ether (ETBE), methyl *t*-butyl ether (MTBE) and *t*-amyl methyl ether (TAME) by *Gordonia terrae*. Appl Microbiol Biotechnol 55:117–121. doi:10.1007/s002530000482. 11234952

[5] Vainberg S, McClay K, Masuda H, Root D, Condee C, Zylstra GJ, Steffan RJ 2006 Biodegradation of ether pollutants by *Pseudonocardia* sp. strain ENV478. Appl Environ Microbiol 72:5218–5224. doi:10.1128/AEM.00160-06. 16885268 PMC1538739

[6] Schuster J, Purswani J, Breuer U, Pozo C, Harms H, Müller RH, Rohwerder T 2013 Constitutive expression of the cytochrome P450 EthABCD monooxygenase system enables degradation of synthetic dialkyl ethers in *Aquincola tertiaricarbonis* L108. Appl Environ Microbiol 79:2321–2327. doi:10.1128/AEM.03348-12. 23354715 PMC3623258

[7] Hyman M 2019 Aerobic degradation of gasoline ether oxygenates, p 389–419. *In* Rojo F (ed), Aerobic utilization of hydrocarbons, oils and lipids. Springer, Cham, Switzerland.

[8] Szabó Z, Gyula P, Robotka H, Bató E, Gálik B, Pach P, Pekker P, Papp I, Bihari Z 2015 Draft genome sequence of *Methylibium* sp. strain T29, a novel fuel oxygenate-degrading bacterial isolate from Hungary. Stand Genomic Sci 10:39. 26221420 10.1186/s40793-015-0023-z PMC4517660

[9] Song L, Florea L, Langmead B 2014 Lighter: fast and memory-efficient sequencing error correction without counting. Genome Biol 15:509. doi:10.1186/s13059-014-0509-9. 25398208 PMC4248469

[10] Magoč T, Salzberg SL 2011 FLASH: fast length adjustment of short reads to improve genome assemblies. Bioinformatics 27:2957–2963. doi:10.1093/bioinformatics/btr507. 21903629 PMC3198573

[11] Schmieder R, Edwards R 2011 Fast identification and removal of sequence contamination from genomic and metagenomic datasets. PLoS One 6:e17288. doi:10.1371/journal.pone.0017288. 21408061 PMC3052304

[12] Edwards R, Edwards JA 2019 fastq-pair: efficient synchronization of paired-end fastq files. bioRxiv. doi:10.1101/552885.

[13] Chevreux B, Wetter T, Suhai S 1999 Genome sequence assembly using trace signals and additional sequence information, p 45–56. *In* Wingender E, Hofestädt R, Giegerich R, Lengauer T, Mewes W, Schomburg D, Vingron M (ed), Computer science and biology: proceedings of the German Conference on Bioinformatics (GCB ’99). GBF-Braunschweig, Department of Bioinformatics, Braunschweig, Germany.

[14] Tatusova T, DiCuccio M, Badretdin A, Chetvernin V, Nawrocki EP, Zaslavsky L, Lomsadze A, Pruitt KD, Borodovsky M, Ostell J 2016 NCBI Prokaryotic Genome Annotation Pipeline. Nucleic Acids Res 44:6614–6624. doi:10.1093/nar/gkw569. 27342282 PMC5001611

[15] Chauvaux S, Chevalier F, Le Dantec C, Fayolle F, Miras I, Kunst F, Beguin P 2001 Cloning of a genetically unstable cytochrome P-450 gene cluster involved in degradation of the pollutant ethyl *tert*-butyl ether by *Rhodococcus ruber*. J Bacteriol 183:6551–6557. doi:10.1128/JB.183.22.6551-6557.2001. 11673424 PMC95485

